# Triggerable RNA nanodevices

**Published:** 2017-06-21

**Authors:** Justin Halman, Emily Satterwhite, Jaclyn Smollett, Eckart Bindewald, Lorena Parlea, Mathias Viard, Paul Zakrevsky, Wojciech K. Kasprzak, Kirill A. Afonin, Bruce A. Shapiro

**Affiliations:** 1 Department of Chemistry, University of North Carolina at Charlotte, 9201 University City Boulevard, Charlotte 28223, North Carolina, USA; 2 Gene Regulation Chromosome Biology Laboratory, Center for Cancer Research, National Cancer Institute, National Institutes of Health, Frederick 21702, Maryland, USA; 3 Basic Science Program, Leidos Biomedical Research, Inc., Center for Cancer Research, National Cancer Institute, National Institutes of Health, Frederick 21702, Maryland, USA

## Abstract

The targeted and conditional activation of pharmaceuticals is an increasingly important feature in modern personalized medicine. Nucleic acid nanoparticles show tremendous potential in this exploit due to their programmability and biocompatibility. Among the most powerful nucleic acid specific treatments is RNA interference-based therapeutics. RNA interference is a naturally occurring phenomenon in which specific genes are effectively silenced. Recently we have developed two different strategies based on customized multivalent nucleic acid nanoparticles with the ability to conditionally activate RNA interference in diseased cells as well as elicit detectable fluorescent responses.[1,2] These novel technologies can be further utilized for the simultaneous delivery and conditional intracellular activation of multiple therapeutic and biosensing functions to combat various diseases.

Nucleic acid nanoparticles have become increasingly attractive in therapeutic research due to their high customizability and various functionalities while retaining biocompatibility and ease in production ^[3–10]^. Specifically, RNA has garnered attention through research exploiting its plethora of functionalities in sensing and treating various diseases. RNA interference (RNAi) which is the effective termination of specific protein production, serves as an important aspect of RNA therapeutics ^[11–13]^. RNAi occurs naturally through various pathways but can be taken advantage of synthetically using a number of designed RNA strands (*i.e*., short-interfering RNA, or siRNA, and slightly elongated dicer-substrate RNA or DS RNA ^[14]^). The ability to simultaneously deliver and activate multiple functionalities (including but not limited to RNAi) would prove immensely beneficial to personalized medicine. Additionally, predicting the structure, folding, and tertiary interactions of RNA would greatly benefit and accelerate the design of therapeutics.

We introduced nucleic acid-based programmable system with the ability to conditionally activate multiple functionalities *via* introduction of split cognate strands ^[15–16]^. Each strand contributes half of a designed DS RNA duplex, which upon re-association and further dicing forms a functional therapeutic. The initially prepared non-functional DNA-RNA hybrids contain either ssDNA or, as in the highlighted work ^[2]^, ssRNA over-hangs of different lengths, called toeholds (Figure 1A). These toeholds allow for the non-functional cognate hybrids to interact and, through branch migration, form more energetically suitable complexes – that is the formation of DS RNA. To allow for multiple functionalities to be delivered and activated simultaneously, previously designed RNA rings ^[17]^ were used in this work as scaffolds carrying six hybrids. The efficacy of re-association based on varying toehold length was assessed using both native-PAGE and FRET. Toeholds of 2, 4, 6, and 8 nucleotides were investigated for their efficiency of re-association. FRET analyses revealed that longer toeholds led to improved re-association rates. The functionality of these particles was confirmed in different human cell lines.

The previously mentioned functionality operates using DNA-RNA hybrids. An additional scheme has also been exploited using RNA-RNA interactions (Figure 1B).^[1]^ In this system an RNA strand is designed to interact with specific mRNA strands in cells. The designed RNA strand contains both “therapeutic” and “trigger” components; these two components are designed to dissociate from each other in the presence of a “trigger” mRNA and form both a byproduct as well as a short hairpin-like RNA which can be processed by dicer to form functional siRNA. The conformational change takes place due to the presence of an extended ssRNA toehold in the “trigger”-binding strand which allows for the specific binding to an mRNA present in diseased cells. This novel approach allows for the conditional activation of therapeutic RNAs only where a designated trigger strand is present. For potential use in treatment of diseases, such as cancer, this can be a tremendous boon as it will reduce off target silencing and allow for more precise treatment. Rational design of these RNA nanoparticles was performed using HyperFold, a novel multistrand nucleic acid modeling approach used for predicting structures and interactions between nucleic acid strands as well as the formation of pseudoknots (RNA structures with non-nested base pairing). This *in silico* approach supplemented *in vitro* experiments to confirm RNA structures and interactions.

The conditionally activated RNA nanoparticles, published back-to-back in Nano Letters [1, 2], offer alternative techniques supplementing each other. The combination of these technologies provides the ability to deliver multiple functionalities simultaneously. Currently there are two synthesis techniques for constructing these architectures and their functional moieties: transcription of individual components followed by a one pot assembly, as is the case for the switch, or co-transcriptional assembly, as in the case of the hybrid nanostructures. The former consists of transcribing DNA strands separately, then purifying and recombining in equimolar concentration by heating (causing denaturation) and snap-cooling to achieve the designed conformation. The latter is a more simplistic approach as it can be done in one step and offers great promise for an alternative pathway to functionalization of particles *in vivo*.

There are other approaches of designed RNA constructs that can switch between different conformations. For example, riboregulators have been developed, that enable protein translation conditional to the binding of a trigger RNA ^[18]^. In a bacterial system, designed riboswitches that are responsive to temperature and binding of a ligand were recently shown to be able to regulate both transcriptional and translational efficiency ^[19]^. Such approaches are not necessarily in competition, but instead have different uses. The two aforementioned approaches are well suited for designing reporter genes or gene circuits but require the de novo design of genomic regions upstream of the translation start site of a gene. The conditional activation of RNA interference (the focus of this research highlight) on the other hand, is based on comparatively short synthetic noncoding RNAs that function in the context of human cancer cells and are thus closer to traditional small molecule therapeutics. Not only will it be interesting to compare these different novel tools for the “smart” control of gene expression: exciting opportunities abound to combine such approaches, leading to systems that allow ever more complex control of the cellular machinery by operating on different levels simultaneously.

Overall, published techniques can be used in conjunction with pre-existing nucleic acid nanostructures thus increasing the control over the formulation and co-delivery of pharmaceuticals and biosensors for the treatment and detection of a large variety of diseases.

## Figures and Tables

**Figure 1.**
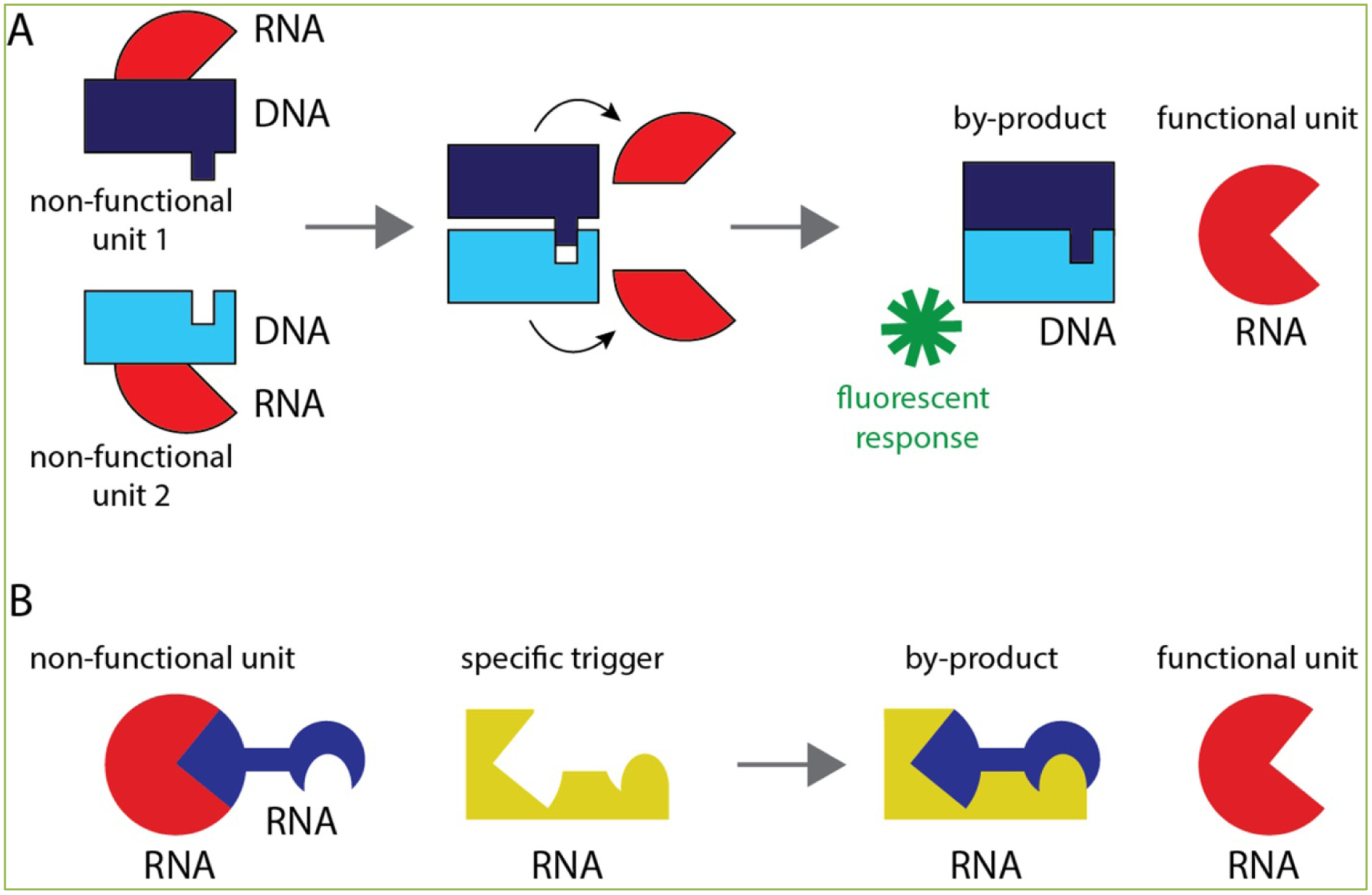
Schematic representation of two alternative ways, described in highlighted papers, to conditionally activate various functionalities intracellularly. **A**: corresponds to the hybrid approach and **B**: corresponds to the two stranded switch approach.
